# Nutritional benefit of fungal spores for honey bee workers

**DOI:** 10.1038/s41598-020-72758-1

**Published:** 2020-09-24

**Authors:** Jorgiane B. Parish, Eileen S. Scott, Katja Hogendoorn

**Affiliations:** School of Agriculture, Food and Wine, The University of Adelaide, Adelaide, SA 5005 Australia

**Keywords:** Ecology, Microbiology, Zoology

## Abstract

The collection of fungal spores by honey bees, *Apis mellifera*, can be classified as active or passive, the latter when spores are associated with pollen, nectar or honey dew. While low quality and shortage of pollen have been raised as hypotheses for fungal spore collection, the nutritional value of fungal spores for honey bees is poorly understood. Here we investigated the effect of consumption of fungal spores on survival, ovarian activation and the development of the hypopharyngeal glands (HPGs) in honey bee workers. Two pollen diets (*Eucalyptus* sp. pollen and a multifloral pollen) supplemented or not with spores of *Botrytis cinerea*, *Cladosporium* sp. or *Colletotrichum acutatum* were used. Consumption of diets that contained fungal spores increased the longevity of honey bee workers but had no significant effect on the development of their HPGs and ovaries. This demonstrates that fungal spores may have nutritional value for honey bees and that the consumption of fungal spores may compensate for nutritional imbalances of poor-quality pollen diets.

## Introduction

Honey bees, *Apis mellifera*, are primary pollinators of crops worldwide^1, 2^ and without these pollinators, the yields of many fruit, seed and nut crops would decrease by more than 90%^3^. Hence, factors that affect bee health, such as the Varroa mite, associated viruses, pesticide use, and nutritional stress, are causes of concern^4–6^. Nutritional stress in honey bee hives can arise either through a lack of floral resources, for example due to drought, urbanisation or planting of crops that do not provide adequate food for bees. While nutrient deficiency can be obvious during pollination events in monocultures^7–9^, even polyfloral scenarios may be composed of combinations of floral resources that are either unhealthy due to the presence of certain toxic components or are lacking in certain amino acids^10^.

Spore collection has been described as ‘incidental’ or ‘the collection in the lieu of pollen’ *(sic)*^11, 12^, where the term ‘incidental’ refers to passive acquisition on the body, while ‘the collection in lieu of pollen’ refers to the occasional active collection by the bees. The use of these terms differs slightly from the historical classification of pollen-collecting behaviour of bees into ‘active’ and’ incidental’^13–18^. In the former case, active pollen collection refers to the purposeful uptake of pollen directly from anthers or other floral surfaces, while incidental pollen collection refers to pollen that accumulates on bees as they forage for nectar. To achieve clarity with regard to the use of these terms, we suggest that both pollen and spore collection be classified as ‘active’ and ‘passive’.

Shaw^11^ suggested that bees might be motivated to collect fungal spores due to (a) lack of floral resources, (b) the chemical composition of spores (i.e. nutrients, amino acids and steroids), (c) attractants such as colour or odour, and (d) because certain spores may resemble pollen grains. However, none of these hypotheses has been experimentally tested. Based on the fact that honey bees actively collect fungal spores as sole load, it has been suggested that the bees might obtain some nutritional benefit from their consumption^11^. Seemingly contrary to this suggestion, detrimental effects of consumption of pure spores of a rust fungus have been experimentally demonstrated^19^. However, bee bread, which is consumed by workers, is unlikely to consist solely of spores, as field-collected spore pellets become mixed with pollen during storage in the hive. Therefore, the health effects of consumption of fungal spores are unclear.

To investigate the functional significance of spore collection, we experimentally tested the effects of consumption of fungal spores in combination with pollen on bee health. We investigated whether diets of pollen plus fungal spores negatively or positively affected survival of workers, ovary activation and development of hypopharyngeal glands (HPGs). Both worker survival and HPG development are related to hive health. HPGs constitute a paired organ, composed of numerous vesicles or acini connected to a duct, in the head of workers^20^. Nurse bees feed the larvae with secretions from these glands^21^. The volume of the acini can serve as an indicator of the nutritional status of a colony^21^, as diets^6^ and fungicides^22^ have been observed to affect HPG development. Similarly, the nutritional value of forage has been linked to ovarian activation^23^. However, the impact of fungal spores on ovarian activation in workers has not been investigated. Here we investigate the effect of consumption of spores of three common fungi, *Botrytis cinerea*, *Cladosporium* sp. and *Colletotrichum acutatum,* in association with *Eucalyptus* sp. pollen (EP) and multifloral pollen (MP) on longevity, the volume of acini in the HPGs and ovarian activation in honey bee workers. These fungi were chosen because honey bee workers have been observed to actively collect spores of *Cladosporium* spp.^24^ and because *Botrytis cinerea* and *Colletotrichum acutatum,* important pathogens of a number of crops, have been demonstrated to be vectored by honey bees^12, 25^.

## Results

### Establishment of the spore concentration in the diet

Honey bee workers did not consume diets solely comprising spores of any of the three species of fungi tested. In addition, workers did not consume diets comprising EP supplemented with spores of *B. cinerea*, *Cladosporium* sp. or *C. acutatum* at weight ratios of 5:1 and 10:1. There was no significant difference in the diet consumption rate among the treatments involving the monofloral EP with or without spores of *B. cinerea, Cladosporium* sp. or *C. acutatum* at the ratio of 20:1 (F = 2.34, *df* = 3, *P* = 0.07, Table S1). Diet consumption rates during the 7-day experiments in which the effects on the hypopharyngeal glands and ovaries were assessed, were similar among the treatments (F = 0.005, *df* = 3, *P* = 0.99). There was no significant interaction between treatments and cages. Hence, as the ratio of 20:1 of pollen to spores allowed assessment of the effects of spore consumption in association with EP, it was used for further assessments.

### Effects of spore consumption on the survival of workers

**Figure 1:**
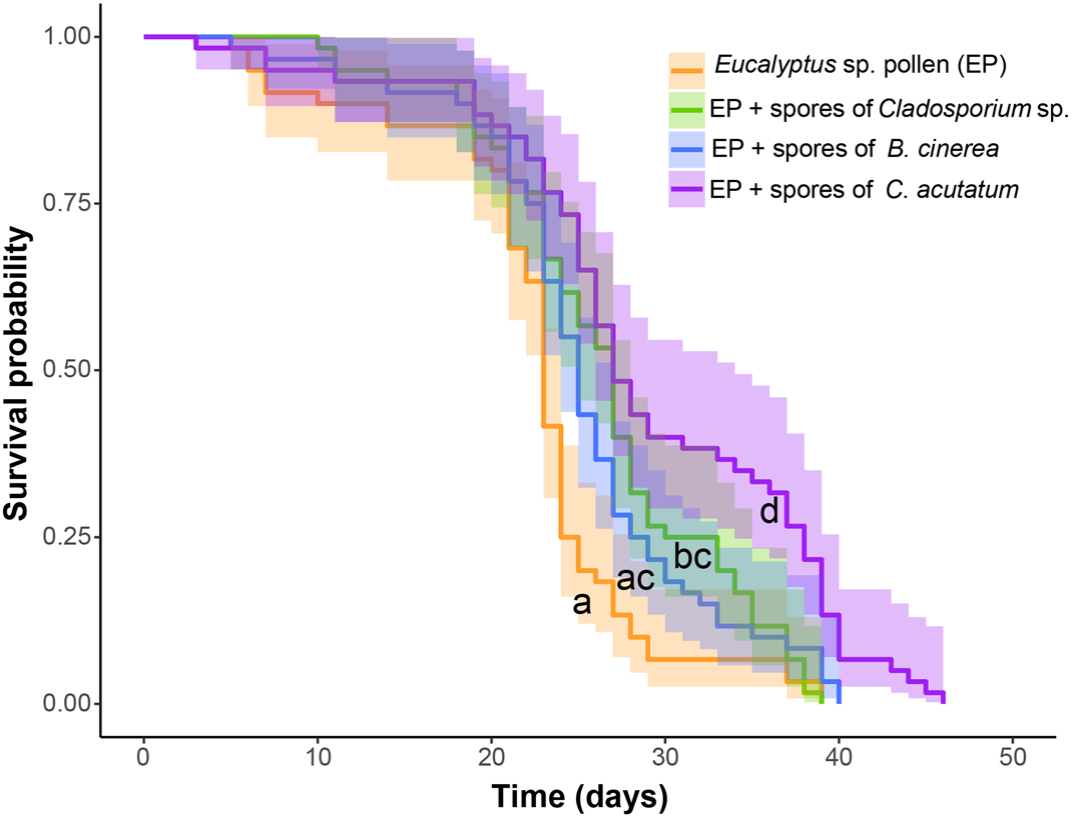
Survival probability of honey bee workers fed *Eucalyptus* sp. pollen alone or supplemented with spores of *Botrytis cinerea*, *Cladosporium* sp. or *Colletotrichum acutatum* at a mass ratio of 20:1 (Mean age at death ± standard error for the treatments in the above order were: 22.43 ± 0.90, 25.31 ± 0.92, 26.5 ± 0.89 and 29.1 ± 1.21). Shaded areas represent 95% confidence intervals. Lower case letters indicate significant differences among the diets (Kaplan–Meier method, log-Rank test *P* < 0.05 and Bonferroni correction).

In an initial experiment in which several ratios of pollen to spores were examined, the survival curves for the workers fed with the monofloral EP with and without fungal spores differed significantly among the treatments (log-rank test and Bonferroni correction: X^2^ = 32, *df* = 3, *P* < 0.05; Fig. 1, Table 1). Workers fed on EP as the sole diet died sooner than bees that consumed the mixture of pollen and fungal spores. Overall, the maximum survival of the bees that consumed EP only, or EP supplemented with spores of *B. cinerea, Cladosporium* sp. or *C. acutatum* was 39, 40, 39 and 46 days, respectively.

**Table 1:** *P* values of the survival analysis for both experiments by the Kaplan–Meier method, log rank test and Bonferroni correction.

	EP	EP + spores of *B. cinerea*	EP + spores of *Cladosporium* sp.	EP + spores of *C. acutatum*	MP	MP + spores of *B. cinerea*	MP + spores of *Cladosporium* sp.
*Eucalyptus* sp. *pollen only*							
EP + spores of *B. cinerea*	n.s	–	–	–	–	–	–
EP + spores of *Cladosporium* sp.	0.02	n.s	–	–	–	–	–
EP + spores of *C. acutatum*	2.5 × 10^–6^	0.01	0.02	–	–	–	–
*Eucalyptus* sp. *and multifloral pollen*							
EP + spores of *B. cinerea*	0.001	–	–	–	–	–	–
EP + spores of *Cladosporium* sp.	4.2 × 10^–5^	n.s	–	–	–	–	–
EP + spores of *C. acutatum*	0.002	n.s	n.s	–	–	–	–
MP	n.s	n.s	0.0005	0.03	–	–	–
MP + spores of *B. cinerea*	n.s	n.s	0.004	n.s	n.s	–	–
MP + spores of *Cladosporium* sp.	0.005	n.s	n.s	n.s	n.s	n.s	–
MP + spores of *C. acutatum*	0.01	n.s	n.s	n.s	n.s	n.s	n.s

EP = *Eucalyptus* sp. pollen, MP = Multifloral pollen, n.s. – Not significant.

**Figure 2:**
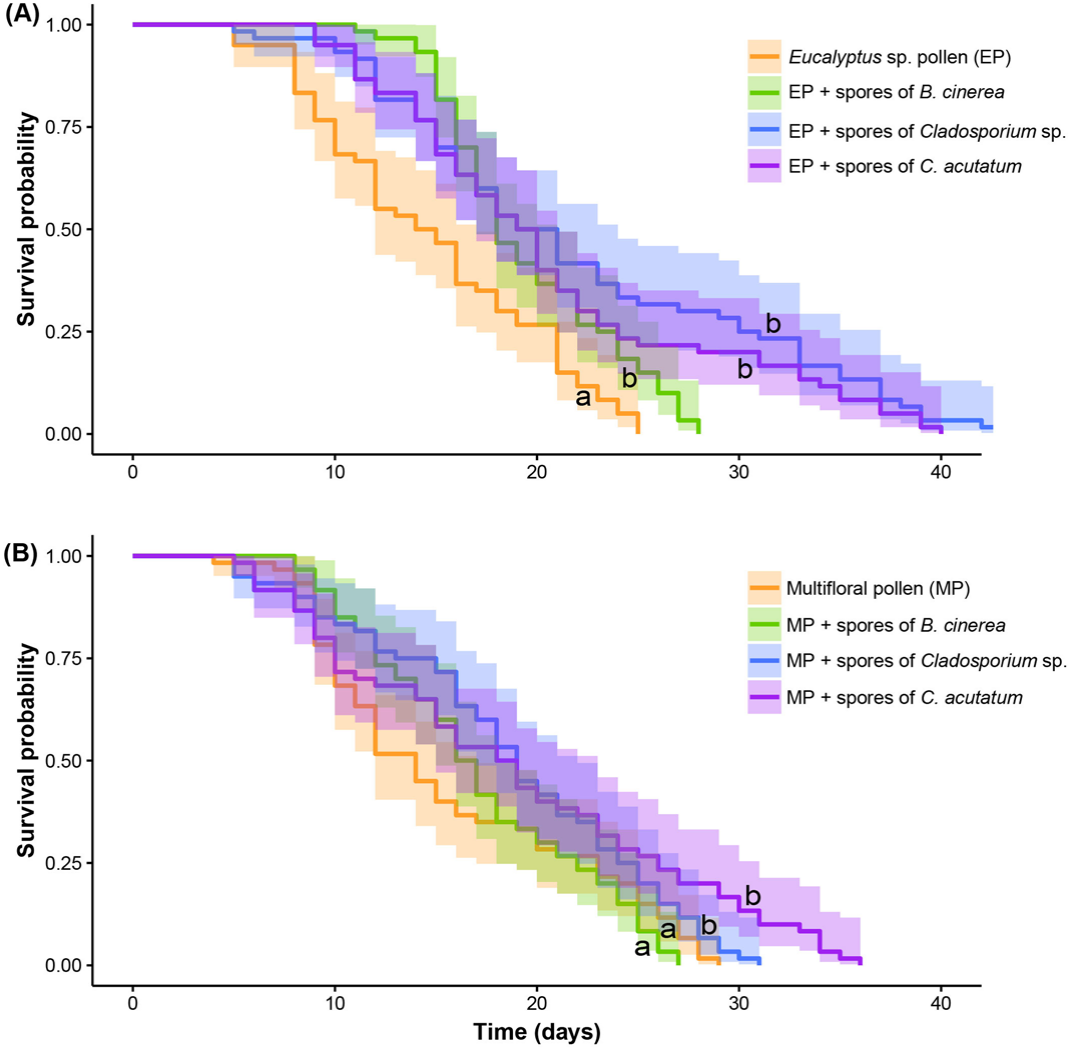
Survival of honey bee workers fed on pollen of *Eucalyptus* sp. (**A**) or multifloral sources (**B**) with or without spores of *Botrytis cinerea*, *Cladosporium* sp. or *Colletotrichum acutatum* at the mass ration of 20:1 (Mean age at death ± standard error for the treatments in the above order were: 14.80 ± 0.74, 19.55 ± 0.56, 22.2 ± 1.25 and 20.8 ± 1.09, 15.81 ± 0.89, 17.1 ± 0.71, 18.66 ± 0.90, 18.78 ± 1.15). Shaded areas represent 95% confidence intervals. Lower case letters indicate significant differences between the diets (Kaplan–Meier method, log-Rank test *P* < 0.05 and Bonferroni correction).

Similar results were obtained in the subsequent comparative experiment using the two pollen diets (i.e. EP and MP) with or without spores of *B. cinerea, Cladosporium* sp. or *C. acutatum.* The survival curves differed significantly between the treatments (log-rank test and Bonferroni correction: X^2^ = 53.8, *df* = 7, *P* < 0.05; Fig. 2; Table 1). Survival of workers that were fed with the pollen-only diets was significantly shorter than of those that consumed the mixtures of pollen and fungal spores, except for the combination of the MP with spores of *B. cinerea*. Overall, the maximum survival of workers that were fed EP only and EP supplemented with spores of *B. cinerea, Cladosporium* sp. or *C. acutatum* was 25, 28, 43 and 40 days, respectively. The maximum survival of workers that were fed MP only and MP supplemented with spores of *B. cinerea, Cladosporium* sp. or *C. acutatum* was 29, 27, 30 and 36 days, respectively. In most cases, the survival of bees that were fed MP was shorter than the bees fed on EP. This corresponds with the generally poorer nutritious value of the multifloral pollen (Table 2). There were no significant differences among the treatments: EP, MP and MP supplemented with spores of *B. cinerea*; EP with spores of *B. cinerea* and all of the diets with the exception of EP alone; EP supplemented with spores of *Cladosporium* sp., MP with spores of *Cladosporium* sp. and MP with spores of *C. acutatum*; EP supplemented with spores of *C. acutatum*, MP supplemented with spores of either *B. cinerea*, *Cladosporium* sp. or *C. acutatum;* or MP supplemented with spores of either *B. cinerea*, *Cladosporium* sp. *C. acutatum*. There was no significant difference among the diets involving the EP and the MP with or without fungal spores in terms of amount consumed per bee (F = 0.91, *df* = 7, *P* = 0.49, Table S1).

**Table 2:** Nutritional composition and amino acid profile of the pollen samples (mean ± standard deviation).

Composition (g/100 g)	Pollen diet	
	*Eucalyptus* sp.	Multifloral
Ash	2.55 ± 0.13	1.91 ± 0.10
Carbohydrates	61.20 ± 3.06	64.40 ± 3.22
Fat	5.30 ± 0.33	6.80 ± 0.42
Protein	28.60 ± 0.92	20.70 ± 0.66
Sodium	14.00 ± 0.35	14.00 ± 0.35
Sugars	39.00 ± 4.29	38.00 ± 4.18
Alanine	1.11 ± 0.00	0.81 ± 0.06
Arginine	1.80 ± 0.04	0.85 ± 0.05
Aspartic acid	2.25 ± 0.00	1.84 ± 0.13
Glutamic acid	2.72 ± 0.02	1.82 ± 0.12
Glycine	1.03 ± 0.00	0.71 ± 0.05
Histidine	0.71 ± 0.01	0.51 ± 0.04
Isoleucine	1.02 ± 0.00	0.73 ± 0.05
Leucine	1.74 ± 0.00	1.25 ± 0.09
Lysine	1.39 ± 0.01	1.05 ± 0.08
Methionine	0.45 ± 0.01	0.33 ± 0.02
Phenylalanine	1.06 ± 0.01	0.74 ± 0.05
Proline	3.20 ± 0.01	1.85 ± 0.13
Serine	1.05 ± 0.01	0.78 ± 0.06
Threonine	0.89 ± 0.00	0.66 ± 0.05
Tyrosine	0.53 ± 0.03	0.39 ± 0.03
Valine	1.22 ± 0.00	0.85 ± 0.05

### Effects of diets on the development of HPGs and ovaries

Neither the volume of the acini in the HPGs nor the ovary activation of workers that consumed EP only or EP supplemented with spores of *B. cinerea*, *Cladosporium* sp. or *C. acutatum* differed significantly among the treatments (HPGs—F = 0.24, *df* = 3, *P* = 0.26, Fig. S1; ovaries—F = 1.66, *df* = 3, *P* = 0.17). For all treatments, the acini appeared normal in shape.

### Gamma irradiation and nutritional factors of pollen samples

The plated suspensions of gamma-irradiated pollen did not yield any microorganisms, whereas non-irradiated monofloral and multifloral pollen yielded approximately 10^5^ CFU of viable fungi/mL of suspension (i.e. gram of pollen) and sporadic bacterial colonies. The nutritional content of the pollen samples (i.e. amino acids, ash, lipids and protein) and the microorganisms naturally associated with them varied between the pollen samples, except for carbohydrates, sugar and sodium levels (Table 2). Pesticide residues were not detected in the samples (Table S2).

## Discussion

Our results demonstrate that consumption of spores of *B. cinerea*, *Cladosporium* sp. and *C. acutatum* in association with pollen can extend the lifespan of honey bee workers but had no effect on the volume of the acini in the HPGs or activation of the ovaries. HPG development and ovary activation are known to be influenced by the quality and quantity of protein ingested by honey bee workers^6, 21, 26, 27^. Although fungal spores generally have a low protein content^28^, spores could conceivably serve as a source of additional nutrients for bees when combined with pollen. This is supported by our data, as bees that fed on pollen supplemented with spores survived longer than those that did not. In addition, the diets with fungal spores contained 5% less pollen than the EP-only diet. As the development of HPGs and ovary activation were similar in diets that did and did not contain spores, the nutritional value of spores may be equal to the value of the EP used in our experiments. It would have been informative to investigate whether spore consumption had a positive effect on these traits in combination with MP. Unfortunately, we did not assess this, as, we did not, a priori, expect the MP to be less nutritious than the EP. The differences between the first and subsequent experiments with respect to the survival of honey bee workers that were fed EP may be due to condition or genetic variation between worker cohorts^29^.

Pollens of different plant species vary greatly in protein content, amino acid composition, lipid, starch, vitamin and mineral content. This can affect individual bee health and colony longevity, physiology, and resistance to or tolerance of disease^6, 30, 31^. The quality of pollen for bees can be classified^32^ according to their levels of crude protein (c.p.) as excellent (> 25% c.p.), average (20–25% c.p.), and poor (< 20%). Our finding that EP was richer in c.p. than the MP was unexpected, but it may explain the finding that workers fed with EP diets typically survived longer than those on the MP diets.

Furthermore, the MP contained the species *Arctotheca calendula* and *Echium plantagineum* which belong to the families Asteraceae and Boraginaceae, respectively. The pollen of some species of the Asteraceae and Boraginaceae contains pyrrolizidine alkaloids^33, 34^, plant secondary compounds which confer protection from herbivory^35^. These alkaloids possess chemical properties that interfere with their digestion by non-specialized bees, including *A. mellifera*^36^. Thus, if pyrrolizidine alkaloids were present in the pollen of *A. calendula* and *E. plantagineum* this might, in part, explain the difference in survival rates between EP and MP. Nevertheless, in both experiments, the survivorship of workers was, overall, extended by the consumption of spores of *B. cinerea*, *Cladosporium* sp. or *C. acutatum* combined with pollen. The consumption of spores of *Cladosporium* sp. and *C. acutatum* in association with either pollen sample used in our experiments provided the longest lifespan, substantiating the hypothesis that nutritional benefits can be obtained by the collection of fungal spores by honey bees.

In addition to the nutritional contribution of pollen and fungal spores tested to the health and survival of honey bee workers, the microorganisms naturally present in the pollen samples, i.e. fungal spores and bacterial cells, could be a source of nutrients that enriches the quality of the diets consumed by the bees. Our results highlight the importance of knowing the composition of the microbiota associated with food sources fed to bees to understand their role and impact on the health and lifespan of bees. Thus, in our gamma-irradiated diets these dead microorganisms may have played a role in the nutritive value of the pollen, a factor that has so far been overlooked in other honey bee feeding experiments^6, 20, 25^. However, it was not possible to determine the nutrient composition of the spores of the three fungi used to supplement the pollen diets due to the challenges of producing sufficient quantities of spores for experimentation and limited resources.

During our experiments, honey bees failed to consume a diet consisting solely of spores of any of the three species of fungi used, nor did they consume spores of the cereal rust fungus, *Puccinia graminis* (data not shown). Comparable results have been reported by Schmidt et al.^19^, in that honey bee workers in cages consumed negligible amounts of spores of *Uromyces* sp. However, in contrast to our experiments, even those small amounts of spores of *Uromyces* sp. were toxic, causing rapid mortality. When spores are collected by bees in the field, it is likely that they are mixed with pollen from different sources and various microorganisms during the formation of bee bread. This would dilute the risk of any possible toxic effects and could enrich the nutritional quality of the food stored in the hives.

Honey bees have been observed to actively collect spores of *Cladosporium* sp.^37^, and our results show that these spores can significantly increase the longevity of the workers. Cell walls of *Cladosporium* sp. contain β-1,3–1,6-glucan, a member of β-D-glucose polysaccharides^37, 38^ which are known to be involved in the immune stimulatory activity in many vertebrate and invertebrate species^39^. Although the effects of β-glucan depend on the target organism and the dose administered^39^, it has been shown to extend the lifespan of honey bee workers^40^. In addition, extracts of the mushroom *Agaricus brasiliensis*, which is rich in glucans*,* have been shown to slightly increase the strength of experimentally fed honey bee colonies^41^. Hence, the β-glucans contained in the cell walls of higher fungi, including *B. cinerea* and *C. acutatum*, might have triggered immune responses in the workers that may have contributed to increasing their survival.

In summary, this study provides evidence that consumption of spores of some fungal species can extend the lifespan of honey bee workers, presumably by providing nutritional benefits. This supports the notion that foraging honey bee workers are likely to actively collect fungal spores. Further research is needed to identify specific interactions between honey bees and different species and strains of fungi; and to investigate the impact of the consumption of fungal spores on the learning, memory, resistance to and tolerance of diseases of bees. Furthermore, it needs to be established whether bee colonies preferentially collect fungal spores when other nutrient sources are in low supply, and whether β-glucans are responsible for the extended longevity of the workers.

## Material and methods

### Pollen samples

Two pollen mixtures were used in the experiments. The first pollen sample was composed mainly of *Eucalyptus* sp. and was collected in Nannup, Western Australia. During the collection of this pollen the main species in flower was *E. baxteri*. The second pollen sample was collected in Adelaide, South Australia, and consisted of a mixture of *Arctotheca calendula* (Cape weed), *Eucalyptus* sp., *Echium plantagineum* (salvation Jane) and *Oxalis pes-caprae* (soursob). Pollen grains were morphologically identified by the acetolysis method described by Erdtman^42^ and glycerine slides were prepared as described in Kearns and Inouye^43^. Photographs showing polar and equatorial views of the pollen grains at 400 × magnification were obtained using a Leica DM750 optical microscope coupled to an ICC50W camera (Leica, Germany) and compared with the APSA^44^ reference key. Both EP and MP samples were obtained from the same beekeeper by placing a pollen trap in front of the hive entrances, subsequently dried and stored at − 20 °C after arrival at the laboratory.

### Gamma irradiation and nutritional quality of pollen samples used in the experiments

Pollen samples were sterilised by subjecting them to gamma irradiation of 25 KGy^45, 46^ at Steritech (Dandenong, Victoria). To ascertain effectiveness, the pollen samples were plated before and after irradiation. For each pollen sample, 1 g was diluted in 0.9 ml of sterile water containing 0.01% (v/v) Tween 20, the suspension was mixed by vortex and a tenfold serial dilution was performed. After mixing each dilution by vortex for 1 min, aliquots of 0.1 ml of the dilutions 1:100 and 1:1,000 were spread onto three replicate plates of potato dextrose agar (PDA, Difco) in 9-cm diameter Petri dishes and incubated at 25 °C under white fluorescent light with 12-h photoperiod. Observations were made 36 h post-plating to determine the number of colony forming units (CFU).

Nutritional quality and pesticide residues of the pollen samples were tested at Agrifood Technology (Werribee, Victoria). Duplicates of each pollen sample was assessed for ash, carbohydrate, lipid, protein, sodium and sugar contents. The protein (N × 6.25) content was determined by the Kjeldahl method^47^ and total lipids were analysed after the disruption of pollen walls by hydrolysis with hydrochloric acid. The presence of pesticide residues in the two pollen diets was assessed via gas and liquid chromatography with a limit of quantification of 0.01 mg/kg and a limit of detection of 0.005 mg/kg. Amino acid profiles were determined by analysis of duplicate samples at the Australian Proteome Analysis Facility (Sydney, New South Wales).

### Fungal material and spore production

Three fungal species were used in the bioassays: (i) *Botrytis cinerea* (CBS140599, Filamentous Fungi Database, Westerdijk Fungal Biodiversity Institute, Utrecht, The Netherlands, GenBank accession number: KX710078 which corresponds to the 18S ribosomal RNA gene, partial sequence; internal transcribed spacer 1, 5.8S ribosomal RNA gene and internal transcribed spacer 2, complete sequence; and 28S ribosomal RNA gene, partial sequence); (ii) *Colletotrichum acutatum* (EU670080, European Molecular Biology Laboratory, which corresponds to the *Glomerella acutata* strain CSL-1689 internal transcribed spacer 1, partial sequence; 5.8S ribosomal RNA gene, complete sequence; and internal transcribed spacer 2, partial sequence) and (iii) *Cladosporium* sp. (CBS146925, Filamentous Fungi Database, Westerdijk Fungal Biodiversity Institute, Utrecht, The Netherlands, GenBank accession number: MK402112 which corresponds to internal transcribed spacer 1, partial sequence; 5.8S ribosomal RNA gene, complete sequence; and internal transcribed spacer 2, partial sequence). These fungal species had been isolated from grapes, almond, and bee bread, respectively. The first two isolates were obtained from the Plant Pathology laboratory and the third from a honey bee hive located at the School of Agriculture, Food and Wine, University of Adelaide, South Australia, Australia. The *Cladosporium* isolate, which was not identified to species level, shares 100% sequence similarity with *C. cladosporioides*, *C. halotolerans*, *C. sphaerospermum*, and *C. parahalotolerans*^48, 49^. Mycelia were cultured on PDA in 9-cm diameter Petri plates for 10 to 14 days and incubated as described above. Cultures were established in consecutive weeks to generate spores of similar age.

Spores were harvested by flooding 10–14 day-old cultures with 15 mL of sterile reverse osmosis (RO) water containing 0.01% (v/v) Tween 20 and dislodging spores from the mycelia using a sterile plastic spreader. The resulting suspensions were filtered through two layers of sterile cheesecloth to remove hyphal fragments^50^. Spore suspensions from three plates of the same fungus were combined in a sterile 50-mL tube and then centrifuged at 4,500 rpm, 25 °C for 10 min. The supernatant was removed and the remaining suspensions (approximately 0.3–0.4 mL) were placed in sterile lids of 2 mL cryogenic tubes (Simport, Canada) in a biohazard laminar flow cabinet and left to dry overnight. Spores in lids were kept in sterile sealed Petri dishes at 4 °C until diet preparation.

### Bee rearing and feeding

Experiments were conducted with newly emerged workers collected from three hives during March of 2018 and 2019. Colonies were maintained in a bee enclosure located at the Waite Campus, University of Adelaide and were regularly inspected for symptoms of diseases. Workers were obtained by placing single frames with late stage pupae in an incubator at 34 °C and 50–70% relative humidity^6^. The bees emerging from these three frames were collected every 24 h in a jar, mixing them to ensure that any results could not be attributed to genetic differences. From these collections, bees were randomly placed in metal cages (6.5 × 8.5 × 4.5 cm; Small-Life Supplies, England) in groups of 10 individuals and fed with gamma-irradiated pollen and fungal spores offered in a sterile cryogenic lid; and sterile 50% (*w/v*) sucrose solution provided in a 5-mL syringe.

Pollen was mixed with sterile RO water at a mass ratio of 7:3 and subsequently with fungal spores. To establish the optimal concentration of spores, workers were offered spores of each fungus as a sole diet, or in a mix with EP at a weight ratio of 5:1, 10:1 and 20:1. In a subsequent experiment the effects of fungal spores were tested in mixes with EP and MP, mixed separately with spores of each fungus at a mass ratio of 20:1. Diets were freshly prepared every day, measured and provided ad libitum. The rates of diet consumption per bee per day were assessed. At the beginning of the experiments, approximately 10–15 mg of diet per bee per day was offered as in our preliminary experiments workers consumed less than 10 mg of diet on a daily basis. Towards the end of the experiments, the provision of food was adjusted because the workers consumed less than 2 mg per bee per day. Caged bees were incubated as described above. Each treatment consisted of six replications. Dead bees were removed from the cages every day until 100% mortality was reached for every cage.

### Hypopharyngeal gland development and ovary activation

To assess whether fungal spores can affect the development of the HPGs, workers were fed with EP with or without spores of *B. cinerea*, *Cladosporium* sp. or *C. acutatum* at a mass ratio of 20:1. In the same manner as for the longevity experiment, groups of ten newly emerged workers were placed in cages and reared for 7 days. On day 8, bees were placed in 1.5-mL Eppendorf tubes and stored at − 80 °C until dissection. The paired HPG were dissected in 100 μL of physiological saline (0.9% NaCl) and stained with a drop of methyl blue dissolved in Ringer’s solution^20^. Glands were mounted on slides and examined as described above. The development of HPGs was assessed by measuring the length and width of 30 randomly chosen acini per bee using the Leica Acquire 3.4.1 software. The total number of bees assessed for diets of EP, or EP supplemented with spores of *B. cinerea*, *Cladosporium* sp. or *C. acutatum* was 59, 58, 57 and 58, respectively. The volume of each acinus was calculated by applying the following equation: 1/6 × 3.14 x length x width^2^^51^.

The ovaries of five bees per cage were dissected under an binocular dissecting microscope and their development was classified into five stages according to Pernal and Currie^25^ as; 0: undeveloped ovaries, 1: slight enlargement, early stages of differentiation; 2: slight development (presence of distinct cells leading to swellings and constrictions), 3: moderate development (egg volume exceeding that of the nutritive follicle), 4: highly developed (presence of fully formed eggs and ovaries with mature oocytes). The effects on HPG development and ovary activation were investigated for the combination of spores with EP only.

### Statistical analysis

All statistical analyses were executed in R version 3.2.1^52^. The survival data were analysed using the Kaplan–Meier method and log-Rank test. Bonferroni correction was applied to the comparison sets to limit the risk that significant differences would be obtained by chance^53^. The HPG volume data were subjected to analysis of variance followed by Tukey’s honestly significant difference (HSD) test, when appropriate. Diet consumption and ovary activation data were compared using a generalized linear model (GLM) with a Gaussian family distribution and Tukey’s HSD test, when appropriate. Normality and homoscedasticity assumptions were tested by QQ plot and Shapiro test, and by Bartlett test, respectively. Only the acini volume data required transformation [log(x)] prior to analyses of variance.

## Supplementary information

Supplementary file1
