# Supplementary material for: Nutritional benefit of fungal spores for honey bee workers
Source: Sci Rep. 2020 Sep 24;10:15671. doi: 10.1038/s41598-020-72758-1 (PMC7519121; doi:10.1038/s41598-020-72758-1)
Supplement: Supplementary file 1 — Supplementary file1 [file 41598_2020_72758_MOESM1_ESM.pdf]

Revised Title: Nutritional benefit of fungal spores for honey bee workers

Jorgiane B. Parish, Eileen S. Scott, Katja Hogendoorn*

School of Agriculture, Food and Wine, The University of Adelaide, Adelaide, South Australia 5005, Australia

Supplementary Information

Table S1. Daily consumption of the different diets (mean ± standard error) at a mass ratio of 20:1 of pollen and fungal spores during both experiments (Generalized Linear Model).

Diets	Diet consumption (mg/bee/day)	P-value
***Eucalyptus* sp. pollen**		
Eucalyptus sp. pollen (EP)	2.43 ± 0.17	0.07
EP + spores of *B. cinerea*	2.22 ± 0.16	
EP + spores of *Cladosporium* sp.	2.16 ± 0.16	
EP + spores of *C. acutatum*	2.19 ± 0.15	
***Eucalyptus* sp. and multifloral pollen**		
EP	2.65 ± 0.23	0.49
EP + spores of *B. cinerea*	2.39 ± 0.26	
EP + spores of *Cladosporium* sp.	2.17 ± 0.38	
EP + spores of *C. acutatum*	2.18 ± 0.24	
Multifloral pollen (MP)	2.86 ± 0.27	
MP + spores of *B. cinerea*	3.01 ± 0.31	
MP + spores of *Cladosporium* sp.	2.86 ± 0.29	
MP + spores of *C. acutatum*	2.50 ± 0.29	

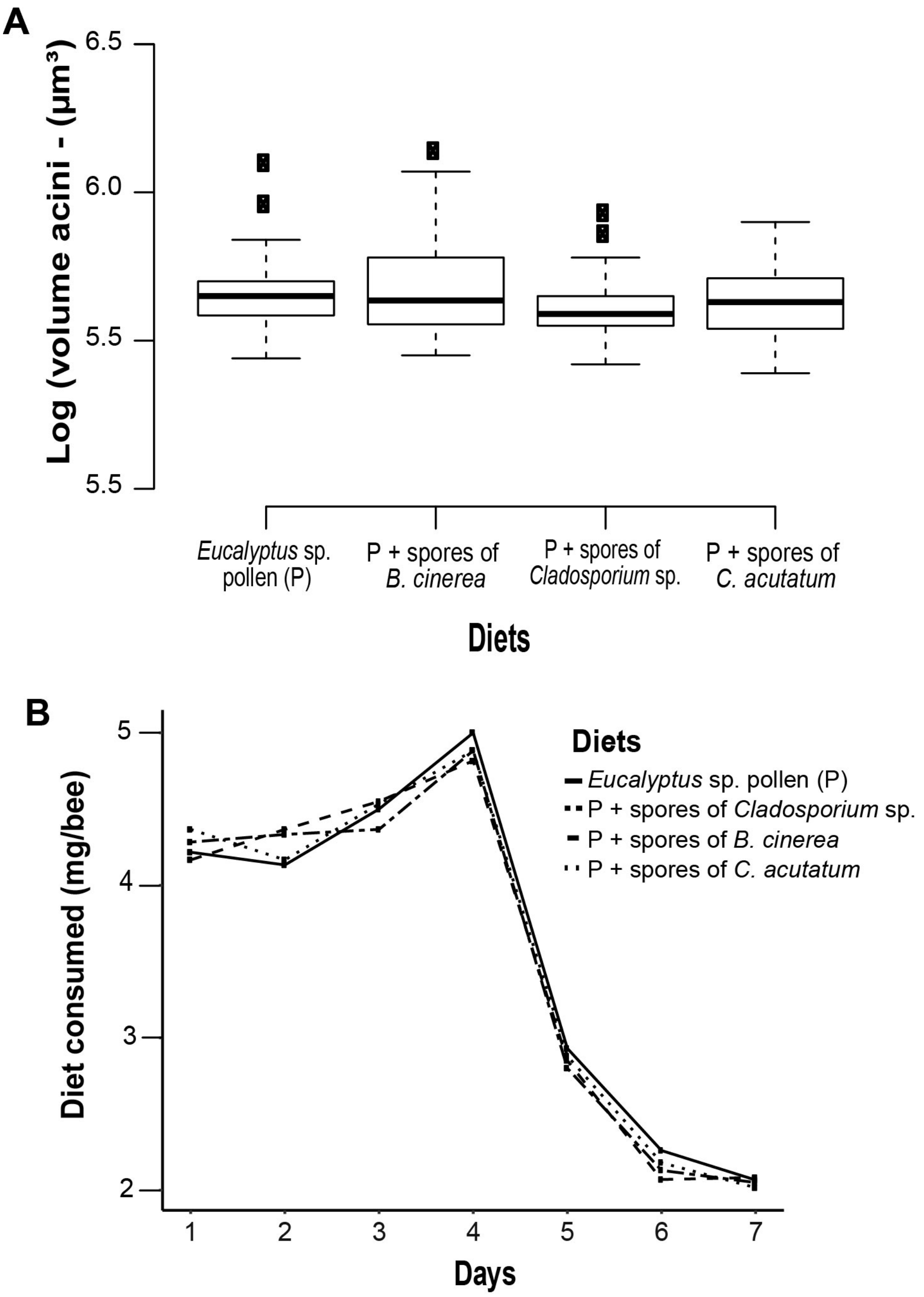

Figure S1. Average volume of the acini of the hypopharyngeal gland **(**A**)** (ANOVA, P>0.05) and (B) diet consumption of workers fed with *Eucalyptus* sp. pollen alone and *Eucalyptus* sp. pollen supplemented with spores of *Botrytis cinerea, Cladosporium* sp. or *Colletotrichum acutatum* at the mass ratio of 20:1 (ANOVA, P>0.05).

Table S2: Concentrations of pesticides detected in pollen samples.

**Pesticides**	*Eucalyptus* sp.	Multifloral
2-phenylphenol (mg/kg)	<0.010	<0.010
Abamectin (mg/kg)	<0.010	<0.010
Acephate (mg/kg)	<0.010	<0.010
Aldrin (mg/kg)	<0.010	<0.010
Atrazine (mg/kg)	<0.010	<0.010
Azinphos methyl (mg/kg)	<0.010	<0.010
Azoxystrobin (mg/kg)	<0.010	<0.010
Benalaxyl (mg/kg)	<0.010	<0.010
BHC alpha (mg/kg)	<0.010	<0.010
BHC beta (mg/kg)	<0.010	<0.010
BHC delta (mg/kg)	<0.010	<0.010
BHC gamma (Lindane) (mg/kg)	<0.010	<0.010
BHC Total (mg/kg)	<0.010	<0.010
Bifenazate (mg/kg)	<0.010	<0.010
Bifenthrin (mg/kg)	<0.010	<0.010
Bioresmethrin (mg/kg)	<0.010	<0.010
Bitertanol (mg/kg)	<0.010	<0.010
Boscalid (mg/kg)	<0.010	<0.010
Buprofezin (mg/kg)	<0.010	<0.010
Captan (mg/kg)	<0.050	<0.050
Carbaryl (mg/kg)	<0.010	<0.010
Carbendazim / Benomyl (mg/kg)	<0.010	<0.010
Chlorantraniliprole (mg/kg)	<0.010	<0.010
Chlordane (mg/kg)	<0.010	<0.010
Chlorfenapyr (mg/kg)	<0.010	<0.010
Chlorfenvinphos (mg/kg)	<0.010	<0.010
Chlorothalonil (mg/kg)	<0.050	<0.050
Chlorpyrifos (mg/kg)	<0.010	<0.010
Chlorpyrifos methyl (mg/kg)	<0.010	<0.010
Chlorthal dimethyl (mg/kg)	<0.010	<0.010
Clofentezine (mg/kg)	<0.010	<0.010
Cyfluthrin (mg/kg)	<0.010	<0.010
Cyfluthrin beta (mg/kg)	<0.010	<0.010
Cyhalothrin (mg/kg)	<0.010	<0.010
Cyhalothrin lambda (mg/kg)	<0.010	<0.010
Cypermethrin (mg/kg)	<0.010	<0.010
Cypermethrin alpha (mg/kg)	<0.010	<0.010
Cyproconazole (mg/kg)	<0.010	<0.010
Cyprodinil (mg/kg)	<0.010	<0.010
DDD p,p (mg/kg)	<0.010	<0.010

Table S2 (continued): Concentrations of pesticides detected in pollen samples.

DDE p,p (mg/kg)	<0.010	<0.010
DDT p,p (mg/kg)	<0.010	<0.010
DDT Total (mg/kg)	<0.010	<0.010
Deltamethrin (mg/kg)	<0.010	<0.010
Diazinon (mg/kg)	<0.010	<0.010
Dichlorvos (mg/kg)	<0.010	<0.010
Dicloran (mg/kg)	<0.010	<0.010
Dicofol (mg/kg)	<0.010	<0.010
Dieldrin (mg/kg)	<0.010	<0.010
Difenoconazole (mg/kg)	<0.010	<0.010
Dimethoate (mg/kg)	<0.010	<0.010
Dimethoate (Total) (mg/kg)	<0.010	<0.010
Dimethomorph (mg/kg)	<0.010	<0.010
Diphenylamine (mg/kg)	<0.010	<0.010
Disulfoton (mg/kg)	<0.010	<0.010
Dithianon (mg/kg)	<0.1	<0.1
Diuron (mg/kg)	<0.010	<0.010
Endosulphan alpha (mg/kg)	<0.010	<0.010
Endosulphan beta (mg/kg)	<0.010	<0.010
Endosulphan sulphate (mg/kg)	<0.010	<0.010
Endosulphan Total (mg/kg)	<0.010	<0.010
Endrin Total (mg/kg)	<0.010	<0.010
Esfenvalerate (mg/kg)	<0.010	<0.010
Ethoprofos (mg/kg)	<0.010	<0.010
Etoxazole (mg/kg)	<0.010	<0.010
Fenamiphos (mg/kg)	<0.010	<0.010
Fenarimol (mg/kg)	<0.010	<0.010
Fenhexamid (mg/kg)	<0.010	<0.010
Fenitrothion (mg/kg)	<0.010	<0.010
Fenoxycarb (mg/kg)	<0.010	<0.010
Fenpyroximate (mg/kg)	<0.010	<0.010
Fenthion (mg/kg)	<0.010	<0.010
Fenvalerate (mg/kg)	<0.010	<0.010
Fenvalerate (Total) (mg/kg)	<0.010	<0.010
Fipronil (mg/kg)	<0.010	<0.010
Flubendiamide (mg/kg)	<0.010	<0.010
Flucythrinate (mg/kg)	<0.010	<0.010
Fludioxonil (mg/kg)	<0.010	<0.010
Flumethrin (mg/kg)	<0.010	<0.010
Flusilazole (mg/kg)	<0.010	<0.010
Fluvalinate (mg/kg)	<0.010	<0.010

Table S2 (continued): Concentrations of pesticides detected in pollen samples.

Fluvalinate tau (mg/kg)	<0.010	<0.010
HCB (mg/kg)	<0.010	<0.010
Heptachlor (mg/kg)	<0.010	<0.010
Heptachlor epoxide (mg/kg)	<0.010	<0.010
Hexaconazole (mg/kg)	<0.010	<0.010
Hexythiazox (mg/kg)	<0.010	<0.010
Imazalil (mg/kg)	<0.010	<0.010
Imidacloprid (mg/kg)	<0.010	<0.010
Indoxacarb (mg/kg)	<0.010	<0.010
Iprodione (mg/kg)	<0.010	<0.010
Kresoxim methyl (mg/kg)	<0.010	<0.010
Linuron (mg/kg)	<0.010	<0.010
Malathion (mg/kg)	<0.010	<0.010
Metalaxyl (mg/kg)	<0.010	<0.010
Methamidophos (mg/kg)	<0.010	<0.010
Methidathion (mg/kg)	<0.010	<0.010
Methomyl (mg/kg)	<0.010	<0.010
Methomyl Oxime (mg/kg)	<0.010	<0.010
Metribuzin (mg/kg)	<0.010	<0.010
Mevinphos (mg/kg)	<0.010	<0.010
Monocrotophos (mg/kg)	<0.010	<0.010
Myclobutanil (mg/kg)	<0.010	<0.010
Omethoate (mg/kg)	<0.010	<0.010
Oxyfluorfen (mg/kg)	<0.010	<0.010
Paclobutrazol (mg/kg)	<0.010	<0.010
Parathion ethyl (mg/kg)	<0.010	<0.010
Parathion methyl (mg/kg)	<0.010	<0.010
Penconazole (mg/kg)	<0.010	<0.010
Pendimethalin (mg/kg)	<0.010	<0.010
Penthiopyrad (mg/kg)	<0.010	<0.010
Permethrin (mg/kg)	<0.010	<0.010
Phenothrin (mg/kg)	<0.010	<0.010
Phorate (mg/kg)	<0.010	<0.010
Phosmet (mg/kg)	<0.010	<0.010
Piperonyl butoxide (mg/kg)	<0.010	<0.010
Pirimicarb (mg/kg)	<0.010	<0.010
Pirimiphos methyl (mg/kg)	<0.010	<0.010
Prochloraz (mg/kg)	<0.010	<0.010
Procymidone (mg/kg)	<0.010	<0.010
Profenofos (mg/kg)	<0.010	<0.010
Propargite (mg/kg)	<0.010	<0.010

Table S2 (continued): Concentrations of pesticides detected in pollen samples.

Propiconazole (mg/kg)	<0.010	<0.010
Prothiofos (mg/kg)	<0.010	<0.010
Pymetrozine (mg/kg)	<0.010	<0.010
Pyraclostrobin (mg/kg)	<0.010	<0.010
Pyrethrins (mg/kg)	<0.010	<0.010
Pyrimethanil (mg/kg)	<0.010	<0.010
Pyriproxyfen (mg/kg)	<0.010	<0.010
Quintozene (mg/kg)	<0.010	<0.010
Spinetoram (mg/kg)	<0.010	<0.010
Spinosad (mg/kg)	<0.010	<0.010
Spirotetramat (mg/kg)	<0.010	<0.010
Sulfoxaflor (mg/kg)	<0.010	<0.010
Tebuconazole (mg/kg)	<0.010	<0.010
Tebufenozide (mg/kg)	<0.010	<0.010
Tebufenpyrad (mg/kg)	<0.010	<0.010
Terbufos (mg/kg)	<0.010	<0.010
Tetradifon (mg/kg)	<0.010	<0.010
Thiabendazole (mg/kg)	<0.010	<0.010
Thiacloprid (mg/kg)	<0.010	<0.010
Thiamethoxam (mg/kg)	<0.010	<0.010
Tolclofos methyl (mg/kg)	<0.010	<0.010
Triadimefon (mg/kg)	<0.010	<0.010
Triadimenol (mg/kg)	<0.010	<0.010
Trichlorfon (mg/kg)	<0.010	<0.010
Trifloxystrobin (mg/kg)	<0.010	<0.010
Vinclozolin (mg/kg)	<0.010	<0.010
